# Implementation of Hepatitis B Screening Into Routine Antenatal Care to Prevent Mother-to-Child Transmission in Rural Western Uganda

**DOI:** 10.1093/ofid/ofad452

**Published:** 2023-08-25

**Authors:** Sahal Thahir, Enid Muhindo, Brian Turigye, Kenneth Kabagambe, Peyton Thompson, Edgar M Mulogo, Ross M Boyce

**Affiliations:** Department of Pediatrics, University of North Carolina at Chapel Hill, Chapel Hill, North Carolina, USA; Peoples Health and Economic Development Organization, Kasese, Uganda; Department of Community Health, Mbarara University of Science and Technology, Mbarara, Uganda; The National Organisation for People Living with Hepatitis B, Kampala, Uganda; Department of Pediatrics, Division of Infectious Diseases, University of North Carolina at Chapel Hill, Chapel Hill, North Carolina, USA; Department of Community Health, Mbarara University of Science and Technology, Mbarara, Uganda; Department of Medicine, Division of Infectious Diseases, University of North Carolina at Chapel Hill, Chapel Hill, North Carolina, USA; Department of Epidemiology, Gillings School of Global Public Health, University of North Carolina at Chapel Hill, Chapel Hill, North Carolina, USA; Carolina Population Center, University of North Carolina at Chapel Hill, Chapel Hill, North Carolina, USA

**Keywords:** HBV, PMTCT, Tenofovir, vertical transmission

## Abstract

In rural Uganda where birth dose vaccination for hepatitis B is not routine, we implemented a pilot program for preventing mother-to-child transmission that effectively identified women with high-risk hepatitis B virus (HBV) infection and started antiviral treatment during pregnancy. Further work is required to enhance antiviral adherence through delivery to ensure effective prevention of vertical HBV transmission.

Globally, hepatitis B virus (HBV) is among the most common causes of chronic hepatitis, affecting >296 million people and contributing to an estimated 820 000 deaths annually from HBV-related liver disease [1]. The African region accounts for nearly a quarter of the global burden of HBV [2]. A high proportion of transmission events in high-prevalence settings, including the African region, are a result of mother-to-child transmission (MTCT) [3]. In the absence of interventions, MTCT occurs in 70%–90% of infants born to mothers with high-risk infection, defined as an HBV viral load ≥200 000 IU/mL, and in 10%–40% of infants born to mothers who do not have high-risk features to eliminate HBV [4, 5]. The components include (1) HBV surface antigen (HBsAg) screening early in pregnancy, (2) risk stratification of HBsAg-positive women (eg, viral load testing), (3) initiation of antiretroviral prophylaxis during pregnancy, and (4) infant immunization, ideally beginning with birth-dose vaccination.

HBV endemicity in Uganda remains high, with HBsAg seroprevalence estimated to be 4.6% in adults and 0.6% in children [6]. Pediatric HBV infection is particularly a problem in Uganda, where birth-dose vaccination has not yet been incorporated into the Expanded Program on Immunization and hepatitis B immunoglobulin remains prohibitively expensive. Instead, efforts to prevent HBV infection are based on administration of a pentavalent vaccine beginning at 6 weeks of age [2]. This strategy, while potentially cost saving, creates a “gap” during which MTCT may occur. Until there is a change in policy, effective efforts at prevention of MTCT (PMTCT) efforts are focused on the identification of women with high-risk HBV infection during antenatal care (ANC) and initiation of antiviral therapy.

Notably, current Ugandan antenatal guidelines recommend screening for HBV at the first antenatal visit [7], but owing to stockouts of HBsAg rapid diagnostic tests (RDTs) and the lack of established management pathways for those who test positive, screening is not routinely performed. However, key tools are available through the public sector, including centralized viral load testing through the Uganda National Health Laboratory Services (UNHLS), locally manufactured antiviral medication (tenofovir [TDF]), and the pentavalent vaccine. Despite this progress, the systems and processes required to deploy these tools in a streamlined and effective manner are not yet established outside of large referral centers. Therefore, the aim of this study was to assess the preliminary feasibility and acceptability of implementing an HBV PMTCT program embedded within routine ANC at rural primary health centers in western Uganda [8].

## METHODS

The project took place at 4 primary health centers located in the Kasese District, a rural region of rugged highland terrain. The facilities—which are staffed by clinical officers, nurses, and midwives but not physicians—provide basic outpatient and antenatal services. Before implementation at each site, we conducted an educational and training session with the clinical staff, after which time study personnel performed a period of direct observation to ensure adherence to protocols.

Pregnant women presenting to their first ANC visit underwent screening with the Standard Q HBsAg RDT (SD Biosensor), which was purchased from local suppliers and has a reported sensitivity and specificity of 100% [9]. Testing was conducted by antenatal clinic staff who had experience performing and interpreting RDTs for other diseases (eg, human immunodeficiency virus [HIV]), and results were provided to participants during the visit. HBsAg-positive women subsequently underwent venous blood sampling for HBV DNA testing, which was available at no cost through UNHLS. Because assessment of renal and liver function is not routinely available, we contracted with a local laboratory to perform testing. We defined “high risk” HBV infection as HBV DNA levels ≥200 000 IU/mL, in accordance with 2020 World Health Organization and local Ugandan guidelines; hepatitis B e antigen testing was not available in this context [7, 10]. Women with high-risk HBV infection were started on TDF prophylaxis beginning at 28–32 weeks’ gestation under the supervision of the clinical officers and nurses in the Antiretroviral Therapy (ie, HIV) Clinic. TDF was continued through 6 weeks post partum, when the first HBV immunization is scheduled to be given. The primary outcomes of this study were the seroprevalence of HBsAg, uptake of HBV screening, and subsequent PMTCT care among pregnant women seeking ANC.

### Ethical Approval and Consent to Participate

Study procedures were approved by the University of North Carolina Institutional Review Board, the Mbarara University of Science and Technology (MUST) Research Ethics Committee, and the Uganda National Council of Science and Technology. Participating individuals provided written informed consent before undertaking any study-related activities.

## RESULTS

Between February 2021 and January 2022, we enrolled 1065 women who subsequently underwent HBsAg testing at the first ANC visit (Table 1). The median age of participants was 25 years (interquartile range [IQR], 16–47 years), and 27.1% of participants were primigravid. The median gestational age at the initial ANC visit was 22 weeks (IQR, 8–36 weeks). Demographic characteristics were generally similar between HBsAg-seropositive and HBsAg-seronegative women.

**Table 1.** Demographic Characteristics of Women Seeking Antenatal Care in the Bugoye Subcounty

Characteristic	Women, No. (%)^a^		
	HBV Negative (n = 1015)	HBV Positive (n = 50)	Total (N = 1065)
Age, median (range), y	25.0 (16.0–47.0)	27.0 (18.0–40.0)	25.0 (16.0–47.0)
Gestational age at screening, median (range), wk	22.0 (8.00–36.0)	22.0 (8.00–36.0)	22.0 (8.00–36.0)
Trimester at presentation			
1st	67 (6.6)	5 (10.0)	72 (6.8)
2nd	671 (66.1)	27 (54.0)	698 (65.5)
3rd	273 (26.9)	18 (36.0)	291 (27.3)
Living children			
No	281 (27.7)	8 (16.0)	289 (27.1)
Yes	733 (72.2)	42 (84.0)	775 (72.8)
Marital status			
Not married	58 (5.7)	2 (4.0)	60 (5.6)
Married/domestic partnership	957 (94.3)	48 (96.0)	1005 (94.4)
Educational level			
None	33 (3.3)	1 (2.0)	34 (3.2)
Primary	673 (66.3)	34 (68.0)	707 (66.4)
Secondary	273 (26.9)	12 (24.0)	285 (26.8)
Tertiary	35 (3.4)	3 (6.0)	38 (3.6)
Religion			
Protestant	481 (47.4)	18 (36.0)	499 (46.9)
Catholic	287 (28.3)	15 (30.0)	302 (28.4)
Pentecostal	71 (7.0)	6 (12.0)	77 (7.2)
Muslim	25 (2.5)	1 (2.0)	26 (2.4)
Other	148 (14.6)	10 (20.0)	158 (14.8)

Abbreviation: HBV, hepatitis B virus.

a Data represent no. (%) of women unless otherwise specified.

All 1065 enrolled women accepted HBsAg testing at their initial antenatal visit. A total of 49 women (4.6%) tested positive for HBsAg. Completion of HBV viral load testing was high, with 47 of 49 HBV-positive women (95.9%) undergoing additional blood sample collection. Forty-two participants had documented titer results from UNHLS; for 5 participants, results were never returned. The median turnaround time from sample collection to receipt of HBV DNA results was 46 days (IQR, 28–73 days).

Among HBsAg-positive women with a HBV viral load result, 4 (10.3%) had viral loads ≥200 000 IU/mL and were categorized as high risk. At baseline testing, none of these women had evidence of significant kidney impairment that might preclude TDF use or require dose adjustment. All high-risk women started TDF therapy by 28 weeks’ gestation, though only 1 continued therapy through our target of 6 weeks post partum.

**Figure 1.**
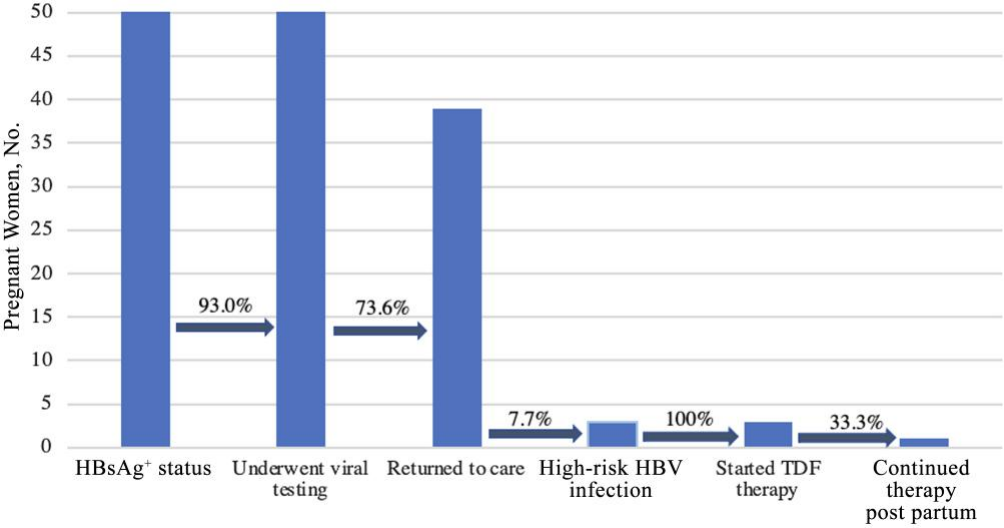
Care cascade for prevention of mother-to-child transmission of hepatitis B virus (HBV) among HBV surface antigen–seropositive (HBsAg^+^) mothers in rural western Uganda. Abbreviation: TDF, tenofovir.

Overall, while acceptance of initial screening was high, we observed that the largest share of attrition in the care cascade occurred in the period immediately after women screened positive for HBsAg. (Figure 1) All women who were screened as high risk had high acceptance of antiviral prophylaxis, though only 2 participants (50% of high-risk cases) had documented TDF adherence through delivery, based on questionnaires at ANC visits. These participants also had follow-up laboratory work that showed appropriately decreasing viral titers during antiviral treatment.

## DISCUSSION

Our findings suggest that routine screening for HBV as part of antenatal services at a primary health facility—the type of setting where most women receive care—was logistically feasible and universally accepted in rural western Uganda. However, challenges remain regarding the evaluation of women who are HBsAg positive, with more than a quarter of women falling out of the care cascade before risk stratification. Among women identified as being at high risk of transmission, the initial acceptance of antiviral prophylaxis was high. Yet rates of completion—albeit in a small sample—were low. Taken together, our early experience, combined with the results of previous trials [11, 12], suggests that with further implementation efforts, embedding PMTCT into routine ANC at peripheral health facilities in rural Uganda, may be a feasible component of HBV elimination.

One key factor that may have contributed to high attrition among enrolled women is the lack of access to clinical laboratory resources across the region, even at many higher-level public health facilities. Within the local region, testing for compete blood count and kidney function is available only in the private sector, which incurs both direct and indirect (eg, travel) costs to patients. While our program covered the direct costs, many patients elected to leave the clinic before a new blood sample could be obtained, which may be indicative of the substantial travel burden required to reach the clinic and/or inadequate education among patients of the importance of further care, a pattern we have observed with HIV care. Given that none of the women with high-risk HBV infection had underlying medical conditions that would have precluded antiviral initiation, it may also be possible to streamline algorithms to limit the testing burden.

There was also substantial delay in turnaround of HBV viral load results. With nearly 50 days between sample collection and return of results—and often no good way to communicate with patients outside of clinic visits—it is likely that some patients were simply lost to follow-up. Furthermore, most women did not present until the second trimester. In some circumstances, the long turnaround period made it challenging to initiate antiviral prophylaxis by the third trimester. One potential intervention to address this barrier would be on-site viral load testing via a commercially available rapid molecular platform already used at higher-level facilities in Uganda for tuberculosis diagnosis. HBV viral load results are typically available in about 90 minutes and could be used to differentiate high-risk women who require additional care in a timelier manner and could also be used to monitor response to treatment after TDF initiation.

In conclusion, our preliminary findings show that screening for HBV infection as part of routine ANC is feasible and highly acceptable in lower-level health facilities, but key gaps remain that could reduce effectiveness. Decentralization of laboratory infrastructure may overcome barriers to timely risk stratification, but more work is needed to understand low rates of antiviral adherence. Additional analyses of the uptake of infant HBV vaccine and subsequent serologic testing of infants at 6 months of age will follow.

## Notes

***Disclaimer.*** The funders had no role in the study conception or design and will not have any role in the analysis of the results.

***Data availability.*** Deidentified individual data will be shared beginning 9–36 months after publication, provided that the investigator who proposes to use the data has approval from an institutional review board, independent ethics committee, or research ethics board, as applicable, and executes a data use/sharing agreement with the University of North Carolina.

All other authors report no potential conflicts.
